# Retinal Thickness in Patients with Parkinson’s Disease and Dopa Responsive Dystonia—Is There Any Difference?

**DOI:** 10.3390/biomedicines13051227

**Published:** 2025-05-19

**Authors:** Marko Svetel, Gorica Marić, Marija Božić, Una Lazić, Andona Milovanović, Jana Jakšić, Igor Petrović, Ana Dimitrijević, Milica Knežević, Tatjana Pekmezović

**Affiliations:** 1 Clinic for Eye Disease, University Clinical Center of Serbia, 11000 Belgrade, Serbia; marija.bozic@med.bg.ac.rs (M.B.); janajaksicmfub@gmail.com (J.J.); ana.m.dimitrijevic@gmail.com (A.D.); knezmima@gmail.com (M.K.); 2 Institute of Epidemiology, Faculty of Medicine, University of Belgrade, 11000 Belgrade, Serbia; gorica.maric@med.bg.ac.rs (G.M.); pekmezovic@orion.rs (T.P.); 3 Faculty of Medicine, University of Belgrade, 11000 Belgrade, Serbia; ulazic99@gmail.com (U.L.); igor.n.petrovic@gmail.com (I.P.); 4 Neurology Clinic, University Clinical Center of Serbia, 11000 Belgrade, Serbia; andona8@gmail.com

**Keywords:** optical coherence tomography, retinal thickness, Parkinson’s disease, dopa responsive dystonia

## Abstract

**Background/Objectives**: Certain aspects of retinal thickness assessed by optical coherence tomography (OCT) in patients with Parkinson’s disease (PD) require additional clarification. It is supposed that attributing reduced retinal thickness in PD to dopaminergic loss may not be acceptable as it also happens in diseases where dopaminergic loss does not occur. The objective of our study is to compare the ganglion cell/inner plexiform layer (GCIPL), peripapillary retinal nerve fiber layer (pRNFL), and macular thickness of PD and dopa responsive dystonia (DRD) patients with healthy controls (HC), to investigate whether DRD patients, as a distinctive model of genetically induced dopamine deficiency, have reduced retinal thickness in comparison with PD, and to analyze correlation between retinal thickness and various PD clinical parameters. **Methods**: We analyzed 86 patients with PD, 10 patients with DRD, and 96 age- and sex-matched HC. **Results**: GCIPL, pRNFL, and central macula thickness (CMT) are statistically significantly thinner in PD patients compared to HC (*p* < 0.001, all). GCIPL and CMT are also statistically significantly thinner in DRD patients compared to HC (*p* = 0.012, *p* = 0.001, respectively). GCIPL thickness correlates positively with the daily dose of levodopa (r = 0.244, *p* < 0.01). The thickness of GCIPL and pRNFL correlate negatively with current age (r = −0.219; *p* < 0.01 and r = −0.358; *p* < 0.05, respectively). All retinal parameters are statistically significantly thinner in females than in males (*p* < 0.05). **Conclusions**: Patients with PD and DRD did not differ in GCIPL and pRNFL thickness when compared to one another. These results, supported by positive correlation of levodopa dose and GCIPL thickness in PD patients, emphasize the importance of dopamine in maintaining retinal thickness.

## 1. Introduction

Optical coherence tomography (OCT), a high-resolution imaging modality, is an invaluable tool for detecting alterations in the retina, the solely in vivo optically accessible component of the central nervous system (CNS). Due to their common embryological origin, retinal analysis offers a unique opportunity to investigate CNS pathology. This method has been used in the past, aiming to enhance understanding of neurodegenerative diseases, such as Parkinson’s disease (PD) [1].

The heterogeneity in the research design complicates the interpretation of results, sometimes leading to contradicting conclusions and comparisons. The duration and severity of the condition vary among study groups, as do cognitive status, selected eyes for assessment, information regarding ophthalmological examinations to identify concurrent visual conditions, and the OCT devices utilized for investigation [2,3,4,5,6,7]. The inconsistency in findings highlights the need for further examination of certain elements of OCT research.

It is supposed that the degeneration of dopaminergic neurons in the macula may lead to diminished OCT measurements in PD. A hallmark of PD is dopamine loss in the nigrostriatal pathways. Furthermore, the same loss of dopamine is observed in the retina, particularly in the fovea, which contains a higher concentration of dopaminergic amacrine cells [8].

Interestingly enough, other neurological diseases have also been associated with the thinning of the retinal nerve fiber layer (RNFL) and the macular layers. Hence, the thinning of the RNFL and macular layers in PD seems to be a non-specific marker of degeneration rather than a finding unique to PD. Attributing it to dopaminergic loss may be inappropriate, given that reductions in RNFL and macular thickness also occur in other conditions where dopaminergic loss is absent.

However, if dopamine deficiency does not account for macular and RNFL thinning, then normal thickness of these structures should be detected in patients with dopamine deficiency. The natural model of this condition is dopa-responsive dystonia (DRD), an extremely rare disorder, with a prevalence of 0.5 per 1,000,000, resulting from a genetically induced enzyme shortage essential for dopamine synthesis, leading to subsequent dopamine impairment [9].

The objective of our study is to determine whether our DRD patients exhibit reduced retinal thickness compared to those with PD. Furthermore, we aim to investigate patients with PD and compare the thickness of their macula, RNFL, and ganglion cell/inner plexiform layer (GCIPL) with those of HC and to analyze the correlation between retinal thickness and various clinical parameters.

## 2. Materials and Methods

This was an observational, cross-sectional study. Patients were recruited from the Department of Neurodegenerative Diseases at the Clinic for Neurology, University Clinical Centre of Serbia (UCCS), between 1 June 2022, and 31 December 2024, and referred to the Clinic of Ophthalmology, UCCS, for ophthalmological evaluation. The Ethics Committee of the Faculty of Medicine, University of Belgrade, approved the study (No. 25/III-18). This study was conducted in accordance with the principles of the Helsinki Declaration. Informed consent was acquired from all patients involved in the study following a comprehensive explanation of the research.

### 2.1. Participants

The first cohort of PD patients comprised 86 individuals (172 eyes). All patients had evaluations by a neurologist specialized in movement disorders. Patients with PD fulfilled the Movement Disorder Society (MDS) clinical diagnostic criteria for clinically established PD [10].

The second group comprised 10 individuals (20 eyes) with DRD, diagnosed based on clinical presentation, levodopa responsiveness, and genetic confirmation tests.

Ninety-six control individuals (192 eyes) were recruited from volunteers attending the Clinic of Ophthalmology for routine visual acuity assessments. The controls consisted of healthy volunteers, matched for sex and age, with no history of ocular problems except for incipient cataract, dry eye, and small refractive errors.

### 2.2. Clinical Assessment

Clinical and demographic data were collected at baseline from patients by questionnaires and interviews conducted by study physicians and the research coordinator.

The variables were current age, sex, initial symptoms, and levodopa equivalent dosage.

The severity of PD was assessed using the Hoehn–Yahr scale [11] and the MDS-Unified Parkinson’s Disease Rating Scale (MDS-UPDRS) [12]. Patients did not discontinue their dopaminergic medication before the examination.

The Mini Mental State Examination scale (MMSE) was utilized to evaluate cognitive status [13]. The daily dose of levodopa was determined using a standardized method [14].

Every patient underwent a comprehensive ophthalmologic assessment, encompassing Snellen best-corrected visual acuity, applanation tonometry (Goldmann), slit-lamp biomicroscopy, gonioscopy, and indirect ophthalmoscopy.

### 2.3. Criteria for Exclusion

The ophthalmological exclusion criteria were as follows: glaucoma patients and suspects, optic nerve cupping of concern, significant refractive errors exceeding −6 diopters in myopia or +6 diopters in hyperopia, any ocular or systemic conditions that may affect OCT analysis (Diabetes Mellitus, Hypertension, Systemic Lupus Erythematosus, etc.), prior intraocular surgery or trauma, and pregnancy. Other neurological disorders, such as multiple sclerosis or Alzheimer’s disease, were also reasons for exclusion.

Several individuals were omitted due to inadequate visual fixation and restricted wheelchair access to the OCT device.

### 2.4. Optical Coherence Tomography Assessment

All patients and controls underwent OCT imaging using a SD-OCT device (RTVue-100, Optovue, Fremont, CA, USA) to measure peripapillary retinal nerve fiber layer (RNFL) thickness and full and inner macular thickness in μm. Retinal measures for both eyes were obtained individually in all participants and incorporated into the statistical analyses.

RNFL thickness was evaluated using a scanning protocol as follows: the area scanned was 4.9 mm centered on the optic disc, the scan pattern was made up of 13 concentric circles with diameter from 1.3 mm to 4.9 mm with 0.3 mm interval and 12 radial lines, the total number of data points was 14,241, and the scan time was 0.55 s.

The macular retinal thickness was measured in nine segments following the Early Treatment Diabetic Retinopathy Study (ETDRS) grid. The fovea center has a diameter of 1 mm, the parafovea ranges from 1 mm to 3 mm in diameter, and the perifovea spans from 3 mm to 5 mm in dimension. Both parafovea and perifovea were individually segmented into four quadrants: superior, inferior, nasal, and temporal.

Data Quality and Inclusion Criteria: Multiple scans were conducted and a quality rating of 40 or higher was selected for analysis.

The same observer evaluated all OCT images.

The key independent variables were overall average RNFL thickness, GCIPL thickness, and full macular thickness across nine segments, encompassing the central macula, parafoveal, and perifoveal regions.

### 2.5. Statistical Evaluation

Descriptive and inferential statistics were employed in data analysis. Continuous variables were characterized by mean and standard deviation, whilst categorical variables were represented by frequencies and percentages. Demographic and clinical features between patients (PD, DRD) and controls were compared using Student’s *t*-test for continuous variables and Chi-square test for nominal data. The association among various factors was assessed using Pearson and Spearman correlation coefficients.

All statistical analyses were conducted utilizing SPSS (Statistical Package for Social Sciences), version 20. *p* value below 0.05 was deemed significant.

## 3. Results

### 3.1. Patients with PD and Healthy Control Subjects

Our study group comprised 86 patients with PD, encompassing 172 eyes. The mean age was 59.2 ± 11.9 years, with an initial age at disease onset of 52.1 ± 12.6 years. Disease duration was 6.7 ± 5.6 years at the time of evaluation. All patients received treatment, with an equal daily dose of levodopa averaging 583.6 ± 390.5 mg, while the dosage of levodopa alone was 388.4 ± 273.4 mg. The clinical characteristics of the patients related to the severity and stage of the disease, and the cognitive status assessed by the MMSE are shown in Table 1.

The thickness parameters of the analyzed retinal structures (GCIPL, average peripapillary RNFL, and macular thickness in nine examined segments) are compared with the HC (Table 2).

GCIPL, peripapillary RNFL, and central macula (M1) are statistically significantly thinner in PD patients (*p* < 0.001, all).

**Figure 1:**
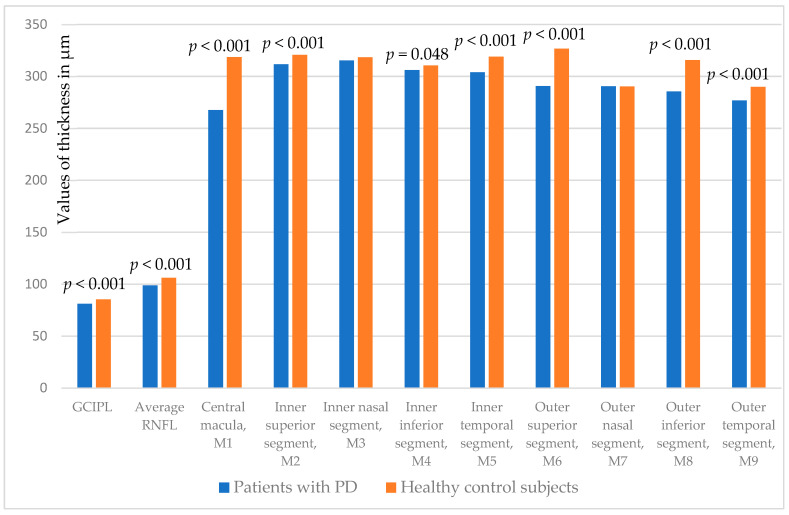
Comparison of GCIPL, peripapillary RNFL, central macula (M1), and additional macula segments thickness (M2-M9) measured at both eyes between PD and healthy control subjects. GCIPL: ganglion cell/inner plexiform layer; RNFL: retinal nerve fiber layer; M1-M9: macular segments according to the Early Treatment Diabetic Retinopathy Study (ETDRS) grid.

Regarding the rest of macular segments (M2-M9), Parkinsonian patients differ from HC in inner superior (*p* < 0.001), inferior (*p* = 0.048), and temporal (*p* < 0.001) segments and outer superior (*p* < 0.001), inferior (*p* < 0.001), and temporal (*p* < 0.001) segments (Figure 1).

The OCT findings of one of our PD patients is presented on Figures S1–S3 (Supplementary File).

The correlation between the observed thinning of the GCIPL, peripapillary RNFL, and the thickness of all macular segments with the clinical and demographic features of individuals with PD is analyzed, as presented in Table 3.

The thickness of GCIPL and RNFL correlate negatively with current age (r = −0.219; *p* < 0.01 and r = −0.358; *p* < 0.05, respectively). GCIPL correlates positively with the daily dose of levodopa (r = 0.244, *p* < 0.01) while RNFL correlates negatively with the same parameter (r = −0.159; *p* < 0.05). Additionally, the worse the UPDRS scores (UPDRS II) and the higher the stage of the disease (HY score) the thicker the GCIPL (r = 0.256, *p* < 0.01 and r = 0.230, *p* < 0.01, respectively).

All assessed retinal segments exhibit a statistically significant reduction in thickness in females compared to males (*p* < 0.05).

In our investigation, there was no correlation between the side of the body initially affected and the thickness of GCIPL, RNFL, and CMT of the ipsilateral or contralateral eye.

### 3.2. Patients with DRD and Healthy Control Subjects

The group of patients with DRD had a mean age of 50.6 ± 13.6 years, and the duration of their illness was 38.6 ± 16.8 years. The initial symptoms manifested at an average age of 11.4 ± 8.3 years. The ratio of affected women to men was 9:1.

They differed from the HC as indicated in Table 4.

**Figure 2:**
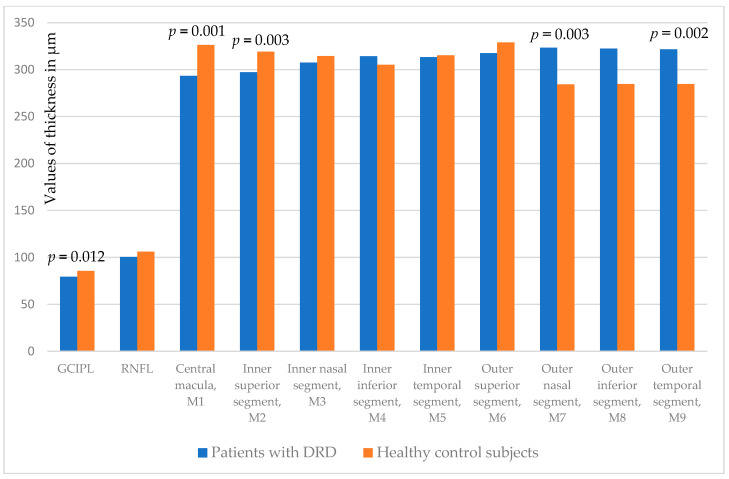
Comparison of GCIPL, peripapillary RNFL, central macula (M1), and additional macula segments thickness (M2-M9) measured in both eyes between DRD and healthy control subjects. GCIPL: ganglion cell/inner plexiform layer; RNFL: retinal nerve fiber layer; M1-M9: macular segments according to the Early Treatment Diabetic Retinopathy Study (ETDRS) grid.

The GCIPL of the patients was statistically significantly thinner compared to the control group (*p* = 0.012) as well as the central macula (*p* = 0.001), inner superior (*p* = 0.003), outer nasal (*p* = 0.003), and outer temporal macular segments (*p* = 0.002) (Figure 2).

### 3.3. Patients with PD and DRD

When compared, it was found that patients with PD and DRD did not differ in GCIPL thickness and RNFL thickness (Table 5).

**Figure 3:**
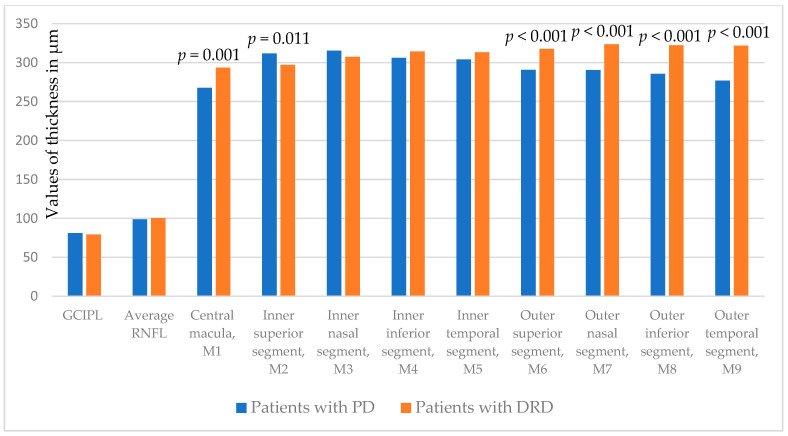
Comparison of GCIPL, peripapillary RNFL, central macula (M1), and additional macula segments thickness (M2-M9) measured at both eyes between PD and DRD patients. GCIPL: ganglion cell/inner plexiform layer; RNFL: retinal nerve fiber layer; M1-M9: macular segments according to the Early Treatment Diabetic Retinopathy Study (ETDRS) grid.

Central macula (*p* = 0.001) and all outer macular segments (*p* < 0.001, all) are statistically significantly thinner in PD patients in comparison with DRD, while superior inner segment (*p* = 0.011) is thicker in PD patients (Figure 3).

## 4. Discussion

### 4.1. Thickness of GCIPL, pRNFL and Macular Segments in PD and DRD Patients

Our cohort demonstrated that GCIPL, pRNFL, and CMT are statistically significantly thinner in the eyes of PD patients compared to HC.

The GCIPL was not thinner in certain studies [15,16], whereas it exhibited a reduction in thickness in others [2,6,17,18,19]. It was determined that these specific characteristics may be useful for assessing neurodegeneration and monitoring neuroprotective therapy [4]. Huang et al. (2021) found that the reduced thickness of the GCIPL and RNFL, along with the lack of variation in the thickness of other retinal layers, aids in distinguishing parkinsonisms, particularly at the disease onset or in their very early stages [3].

Regarding pRNFL, a recently published meta-analysis by Zhou, Tao, and Li (2021) [18] revealed a substantial decrease in mean pRNFL thickness in the PD group, demonstrated by additional researchers as well [3,20,21,22,23]. Contradictorily, other studies have indicated that RNFL thickness is comparable between patients and controls [16,24,25,26,27,28,29].

The central macular thickness in patients with PD differs from that of HC. Zhou, Tao, and Li (2021) demonstrated a substantial decrease in CMT, thickness of all outer macular segments, and macular volume [18]. Bittersohl et al. (2015) asserted that the macular region, particularly the fovea, which possesses the highest concentration of photoreceptor cells, appears to exhibit greater sensitivity and may serve as a possible biomarker [26]. Zhou, Tao, and Li posited that ganglion cells and nerve fibers are absent in the fovea, anticipating that the thickness of the central macula does not differ between patients with PD and controls. The parafoveal and perifoveal macular areas, encompassing a broader area, had reduced thickness [18].

The etiology and pathophysiology of retinal thickness reduction remain ambiguous. A reduction in dopaminergic neurons in the macula may lead to diminished OCT measurements in PD. Post-mortem analyses revealed reduced retinal dopamine concentration in individuals with PD [30]. Ahn et al. are the pioneers in demonstrating the relationship between retinal thickness and dopaminergic depletion in the substantia nigra using Dopamine Transporter Positron Emission Tomography (PET) [31]. Research has demonstrated that dopamine deprivation is associated with the degeneration of amacrine cells in the retina [30]. Huang et al. determined that dopaminergic depletion in PD results in reduced interaction between dopaminergic amacrine cells and retinal ganglion cells, causing atrophy of the ganglion cells and their nerve fibers [3].

Furthermore, immunocytochemical labelling has revealed laminar alpha-synuclein inclusions, a hallmark of PD, in dopaminergic amacrine cells and several retinal layers [32,33]. A recent study by Marrocco et al. involving transgenic mice reveals that the overexpression of alpha-synuclein causes the neurodegeneration of amacrine cells, which subsequently leads to the degeneration of ganglion cells reliant on dopamine support from amacrine cells, ultimately resulting in visual dysfunctions [34].

Toxic alpha-synuclein aggregates at the molecular level result in elevated free radicals, oxidative stress, and mitochondrial impairment, culminating in energy deficit and neurodegeneration. Consequently, a physiologically viable explanation for the observation may be primary dopaminergic degeneration of the inner retina linked to PD due to toxic alpha-synuclein accumulation [35].

An additional hypothesized mechanism contributing to the weakening of retinal structures is vascular involvement; however, researchers discovered no significant difference in retinal vessel diameter between individuals with PD and controls [36,37]. Also, the engagement of sector-specific (temporal) RNFL is a pattern observed in mitochondrial optic neuropathies, indicating mitochondrial dysfunctions in retinal alterations [38].

However, if dopamine insufficiency does not account for the reduction in retinal thickness, normal thickness should be observed in patients whose clinical characteristics are exclusively attributable to dopamine deficiency. The exemplar of this disorder is dopa-responsive dystonia (DRD).

In our cohort of individuals with DRD, GCIPL was considerably thinner compared to the control group. It was observed that individuals with PD and DRD had no differences in GCIPL thickness, suggesting that dopamine may play a pivotal role in preserving retinal thickness.

The central macula is thinner in PD and DRD compared to control subjects, with a notable difference between PD and DRD. Values are statistically considerably lower in PD patients compared to those with DRD. The elucidation of that data may indicate a more substantial and progressive dopaminergic decline in PD compared to patients with DRD, who exhibit more stable and non-progressive symptoms. The dosage of levodopa for treating DRD patients remains consistent throughout the treatment, whereas PD patients require an escalation in dosage.

The RNFL does not distinguish between patients with PD and DRD, while the disparity between DRD and HC approaches significance (*p* = 0.053).

In relation to the macular segments outside the central macula, our PD patients exhibit differences from HC in both the inner and outer superior, inferior, and temporal segments.

The findings of studies examining macular segments were inconsistent. Some studies indicate that the superior outer region was thinner in PD [39], whereas another study found that the inner superior, outer temporal, nasal, and inferior regions were thinner [40]. Furthermore, the inner inferior and temporal regions, as well as the outer inferior and foveal areas, exhibited reduced thickness in another study [41]. Segupta et al. discovered that macular volumes were reduced in both parafoveal and perifoveal regions but not in the central macula [42]. Zhou, Tao, and Li (2021) reviewed fourteen studies and found that the PD group exhibited a substantial decrease in the thickness of the central macula and all outer macular segments when compared to HC [18]. Consequently, findings concerning parafoveal and perifoveal macular segments are predominantly uncertain, as it is in our cohort. Accordingly, we were not able to make a conclusion about patterns of retinal thickness loss.

Concerning macular segments, there are distinctions between DRD and HC in the inner superior, outer nasal, and outer temporal macular segments, whereas PD and DRD differ in the center macula and all outer macular segments. Similar to PD patients, we were unable to develop a model of retinal thinning in the parafoveal and perifoveal segments for DRD patients.

### 4.2. Correlation Between Retinal Thickness Measurements with Different Clinical and Demographic Parameters

#### 4.2.1. Current Age, Sex and Disease Severity

Our findings indicate that GCIPL and pRNFL exhibit a negative correlation with current age, signifying that as patients get older, the GCIPL and pRNFL become thinner. Lee et al. (2014) consistently reported that age is a significant determinant of pRNFL thickness in PD [39]. The annual average pRNFL decline rate was 2.52 µm in PD, significantly exceeding the 1.38 µm observed in the HC group across the 5-year follow-up [43]. No link was observed between the patient’s current age concerning CMT. Subhi et al. (2016) shown that ageing appears to have no effect on the thickness of the central macula [44]. This indicates that CMT is not affected by the age of PD patients, but rather by the disease itself.

Quagliato et al. (2014) asserted that male PD patients had higher retinal layer thickness than females [45]. In our investigation, female PD patients exhibited reduced thickness across all retinal segments, consistent with the referenced paper.

The disease’s severity and the assessment methods employed in the investigations varied significantly, resulting in contentious outcomes.

Our study revealed a positive correlation between the thickness of the GCIPL and disease severity, measured by the HY scale, when analyzing the relationship between retinal thickness measures and disease severity. Unexpectedly, advanced disease indicates an increased thickness of the GCIPL. This may be elucidated by the observation that patients with greater severity of disease are administered with a higher daily dosage of levodopa, which is positively connected with GCIPL thickness in our study.

Correlation was not identified between the average thickness of the RNFL nor the thickness of the central macula. Certain investigations identified no correlation between pRNFL thickness and PD severity, as measured by the modified HY scale and UPDRS I, II, and III, and between pRNFL thickness and the PD duration [6,46,47]. This finding indicated that pRNFL thickness is not an indicator of disease progression but rather of the disease itself. In our investigation, pRNFL in PD patients was lower than in control subjects; nevertheless, this parameter does not correlate with disease severity, indicating that it serves as a marker of disease. Other studies demonstrated inverse relationships between pRNFL thickness and PD severity [48,49], indicating that RNFL may serve as a marker for disease progression. Certain authors unexpectedly identified a positive association between RNFL thickness and UPDRS in the PD cohort [50].

In our group, CMT correlates positively with the UPDRS II score, whereas other researchers have indicated an inverse correlation between macular thickness and UPDRS scores [40].

#### 4.2.2. Cognitive Functions

The MMSE in our study is solely correlated with CMT. These results state that the higher the MMSE, the thicker the central macula. The findings align with a recently published article [51].

While we did not identify a correlation between MMSE and RNFL thickness, many publications have reported a strong positive correlation between both variables [4]. Sung et al. (2019) identified a positive association between GCIPL and cognitive function, as measured by the Montreal Cognitive Assessment score (MOCA) [6].

In healthy adults, retinal RNFL thickness may serve as a predictive biomarker for long-term cognitive decline [52].

It might be that changes in retinal thickness associated with cognitive function in PD patients are not connected only to PD but rather to cognitive impairment in addition.

#### 4.2.3. Affected Side

Our investigation revealed no correlation between the side of the body initially affected and the retinal thickness of the ipsilateral or contralateral eye. Certain investigations [38,53,54] indicated a reduced RNFL thickness on the contralateral side to the more affected body side. Conversely, Matlach et al. observed a reduced RNFL thickness on the ipsilateral eye [47].

#### 4.2.4. Levodopa Daily Dose

Our cohort demonstrated a positive connection between GCIPL and levodopa dose. A greater dosage of levodopa correlates with an increased thickness of the GCIPL. This discovery underscores the significance of dopamine in retinal structure and function.

Unexpectedly, the dosage of levodopa exhibited a negative correlation with the thickness of the RNFL.

Several studies have suggested a protective effect for levodopa based on the assessment of RNFL thickness in users versus non-users [7], although this finding has not been consistently replicated in all research [55]. Furthermore, diminished retinal dopamine levels were observed post-mortem in patients who did not get dopaminergic medication throughout their lifetime, in contrast to those who had such treatment [56,57].

The protective effect of levodopa has been observed in ocular diseases. A comprehensive study involving over 87 million individuals indicated that levodopa users have a reduced likelihood of developing age-related macular degeneration, and if they do get it, the onset was delayed by 8 years [58].

In a separate trial, the administration of levodopa postponed anti-VEGF injection therapy while enhancing visual outcomes in individuals with neovascular age-related macular degeneration [59].

## 5. Conclusions

Patients with PD have thinner GCIPL, peripapillary RNFL, and CMT compared to healthy control subjects.

The cohort of patients with DRD has reduced thickness in the GCIPL and central macula, while the pRNFL thickness approaches statistical significance when compared to healthy control subjects.

Patients with PD and DRD had no differences in GCIPL and RNFL thickness when compared to each other.

These results highlight the importance of dopamine in maintaining retinal thickness. This conclusion is also supported by positive correlation of levodopa dose and GCIPL thickness in PD patients, emphasizing the significance of dopamine in the preservation of retinal ganglion cells.

## Figures and Tables

**Table 1:** Clinical characteristics of PD patients’ group.

	Patients with PD
UPDRS I	5.0 ± 4.9
UPDRS II	8.4 ± 6.3
UPDRS III	26.3 ± 13.6
UPDRS IV	1.0 ± 2.6
Total UPDRS	40.7 ± 22.5
HY stage	I-6
	II-20
	III-35
	IV-15
	V-0
MMSE	28.6 ± 1.8

UPDRS: Unified Parkinson’s disease rating scale; HY stage—Hoehn and Yahr stage of PD; MMSE: Mini mental state examination; ∞-number of patients.

**Table 2:** Comparison of GCIPL, peripapillary RNFL, and macular thickness measured at both eyes between PD and healthy control subjects.

	Patients with PD	Healthy Control Subjects	*p*
GCIPL	81.1 ± 7.2	85.4 ± 7.9	**<0.001**
Average RNFL	98.9 ± 10.0	106.1 ± 7.8	**<0.001**
Central macula, M1	267.7 ± 32.4	318.6 ± 16.6	**<0.001**
Inner superior segment, M2	311.7 ± 23.5	320.6 ± 12.4	**<0.001**
Inner nasal segment, M3	315.2 ± 26.7	318.4 ± 17.8	0.191
Inner inferior segment, M4	306.0 ± 22.1	310.5 ± 19.4	**0.048**
Inner temporal segment, M5	303.9 ± 24.8	319.0 ± 17.2	**<0.001**
Outer superior segment, M6	290.6 ± 23.6	326.7 ± 11.7	**<0.001**
Outer nasal segment, M7	290.5 ± 25.2	290.3 ± 35.7	0.958
Outer inferior segment, M8	285.5 ± 28.2	315.6 ± 15.2	**<0.001**
Outer temporal segment, M9	276.7 ± 26.9	289.9 ± 36.2	**<0.001**

GCIPL: ganglion cell/inner plexiform layer; RNFL: retinal nerve fiber layer; M1-M9: macular segments according to the Early Treatment Diabetic Retinopathy Study (ETDRS) grid.

**Table 3:** Correlation between GCIPL, peripapillary RNFL, and macular segments thickness with clinical and demographic parameters.

	GCIPL	RNFL	M1	M2	M3	M4	M5	M6	M7	M8	M9
Current age	−0.219 *	−0.358 **	−0.042	−0.161 *	−0.186 *	−0.287 **	−0.262 **	−0.290 **	−0.336 **	−0.226 **	−0.079
Levodopa daily dose	0.244 *	−0.159 *	0.016	−0.019	0.077	0.069	−0.001	0.034	0.021	0.025	0.223 **
LEDD	0.175	−0.008	0.147	0.011	0.102	0.068	0.115	0.185 *	0.159 *	0.171 *	0.102
UPDRS I	−0.172	−0.001	−0.050	−0.153 *	−0.057	−0.080	0.009	−0.032	−0.006	−0.021	−0.030
UPDRS II	0.256 *	−0.031	0.153 *	−0.081	−0.033	−0.109	0.014	0.093	0.039	0.128	−0.078
UPDRS III	0.183	−0.012	0.131	−0.144	0.016	−0.076	0.089	0.191 *	0.104	0.301 **	−0.090
UPDRS IV	−0.069	0.064	−0.028	−0.006	−0.062	−0.077	−0.060	−0.066	−0.044	−0.101	−0.022
Total UPDRS	0.143	−0.007	0.108	−0.144	−0.019	−0.103	0.053	0.124	0.065	0.199 **	−0.085
HY	0.230 *	−0.049	0.098	−0.113	0.048	−0.013	0.024	−0.178 *	−0.116	−0.155 *	0.026
MMSE	0.198	0.016	0.171 *	0.124	0.075	0.147	0.081	0.066	0.025	0.033	0.043

* *p* < 0.05; ** *p* < 0.01; LEDD: Levodopa equivalent daily dose; UPDRS: Unified Parkinson’s disease rating scale; HY: Hoehn and Yahr staging rating scale; MMSE: mini mental state examination; M1: central macula; M2: inner superior segment; M3: inner nasal segment; M4: inner inferior segment; M5: inner temporal segment; M6: outer superior segment; M7: outer nasal segment; M8: outer inferior segment; M9: outer temporal segment.

**Table 4:** Comparison of different retinal parameters measured at both eyes between DRD and healthy control subjects.

	Patients with DRD	Healthy Control Subjects	*p*
GCIPL	79.4 ± 7.0	85.6 ± 7.9	**0.012**
RNFL	100.5 ± 10.8	106.1 ± 6.6	0.053
Central macula, M1	293.4 ± 37.8	326.2 ± 12.6	**0.001**
Inner superior segment, M2	297.1 ± 28.4	319.1 ± 7.1	**0.003**
Inner nasal segment, M3	307.4 ± 21.7	314.5 ± 16.1	0.247
Inner inferior segment, M4	314.3 ± 22.0	305.2 ± 26.8	0.247
Inner temporal segment, M5	313.3 ± 15.9	315.1 ± 14.4	0.710
Outer superior segment, M6	317.5 ± 26.8	329.0 ± 13.1	0.091
Outer nasal segment, M7	323.4 ± 23.8	284.3 ± 33.2	**0.003**
Outer inferior segment, M8	322.3 ± 22.2	284.7 ± 30.9	0.583
Outer temporal segment, M9	321.6 ± 21.7	284.7 ± 30.9	**0.002**

GCIPL: ganglion cell/inner plexiform layer; RNFL: retinal nerve fiber layer; M1-M9: macular segments according to the Early Treatment Diabetic Retinopathy Study (ETDRS) grid.

**Table 5:** Comparison of different retinal parameters measured at both eyes between DRD and PD patients.

	Patients with PD	Patients with DRD	*p*
GCIPL	81.1 ± 7.2	79.4 ± 7.0	0.331
Average RNFL	98.9 ± 10.0	100.5 ± 10.8	0.519
Central macula, M1	267.7 ± 32.4	293.4 ± 37.8	**0.001**
Inner superior segment, M2	311.7 ± 23.5	297.1 ± 28.4	**0.011**
Inner nasal segment, M3	315.2 ± 26.7	307.4 ± 21.7	0.208
Inner inferior segment, M4	306.0 ± 22.1	314.3 ± 22.0	0.118
Inner temporal segment, M5	303.9 ± 24.8	313.3 ± 15.9	0.102
Outer superior segment, M6	290.6 ± 23.6	317.5 ± 26.8	**<0.001**
Outer nasal segment, M7	290.5 ± 25.2	323.4 ± 23.8	**<0.001**
Outer inferior segment, M8	285.5 ± 28.2	322.3 ± 22.2	**<0.001**
Outer temporal segment, M9	276.7 ± 26.9	321.6 ± 21.7	**<0.001**

GCIPL: ganglion cell/inner plexiform layer; RNFL: retinal nerve fiber layer; M1-M9: macular segments according to the Early Treatment Diabetic Retinopathy Study (ETDRS) grid.

## Data Availability

The data supporting this study’s findings are available from the corresponding author upon reasonable request.

## Supplementary Materials

The following supporting information can be downloaded at: https://www.mdpi.com/article/10.3390/biomedicines13051227/s1, Figure S1. OCT finding of full macular thickness across nine segments in patient with PD, Figure S2. OCT finding of inner macular thickness (GCIPL + RNFL) across nine segments in patient with PD, Figure S3. OCT finding of peripapillary RNFL thickness in patient with PD.

## Abbreviations

The following abbreviations are used in this manuscript:

CNS	Central nervous system
PD	Parkinson’s disease
OCT	Optical coherence tomography
RNFL	Retinal nerve fiber layer
DRD	Dopa responsive dystonia
GCIPL	Ganglion cell/inner plexiform layer
CMT	Central macula thickness
UCCS	University Clinical Center of Serbia
HC	Healthy controls
MDS	Movement Disorder Society
MDS-UPDRS	MDS-Unified Parkinson’s disease rating scale
MMSE	Mini Mental State Examination
ETDRS	Early Treatment of Diabetic Retinopathy Study
IRL	Inner retinal layer
SPSS	Statistical Package for Social Sciences
HY	Hoehn and Yahr
M1	Central macula
M2	Inner superior segment
M3	Inner nasal segment
M4	Inner inferior segment
M5	Inner temporal segment
M6	Outer superior segment
M7	Outer nasal segment
M8	Outer inferior segment
M9	Outer temporal segment
PET	Positron Emission Tomography
