# Supplementary material for: Retinal Thickness in Patients with Parkinson’s Disease and Dopa Responsive Dystonia—Is There Any Difference?
Source: Biomedicines. 2025 May 19;13(5):1227. doi: 10.3390/biomedicines13051227 (PMC12108739; doi:10.3390/biomedicines13051227)
Supplement: Supplementary file 1 [file biomedicines-13-01227-s001.zip › biomedicines-3598354-supplementary.pdf]

Supplementary Files: OCT findings of one of our PD patients.

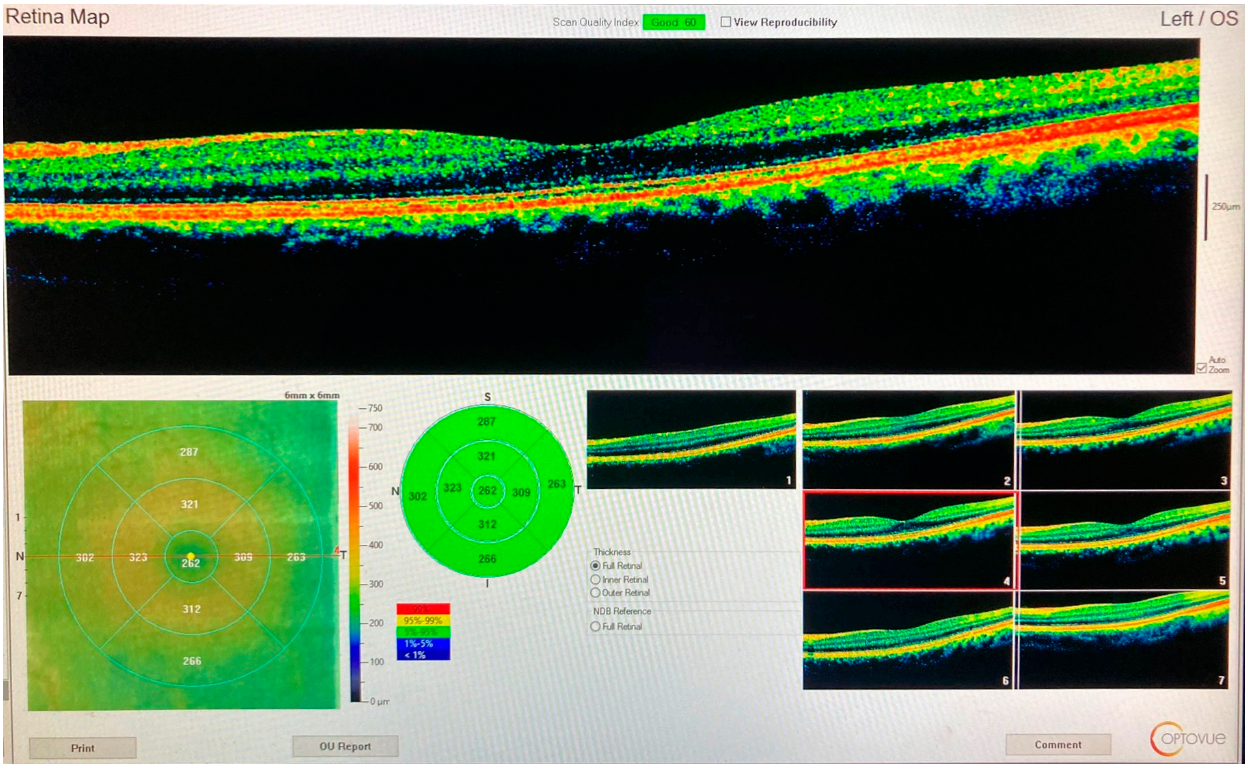

Figure S1. OCT finding of full macular thickness across nine segments in patient with PD.

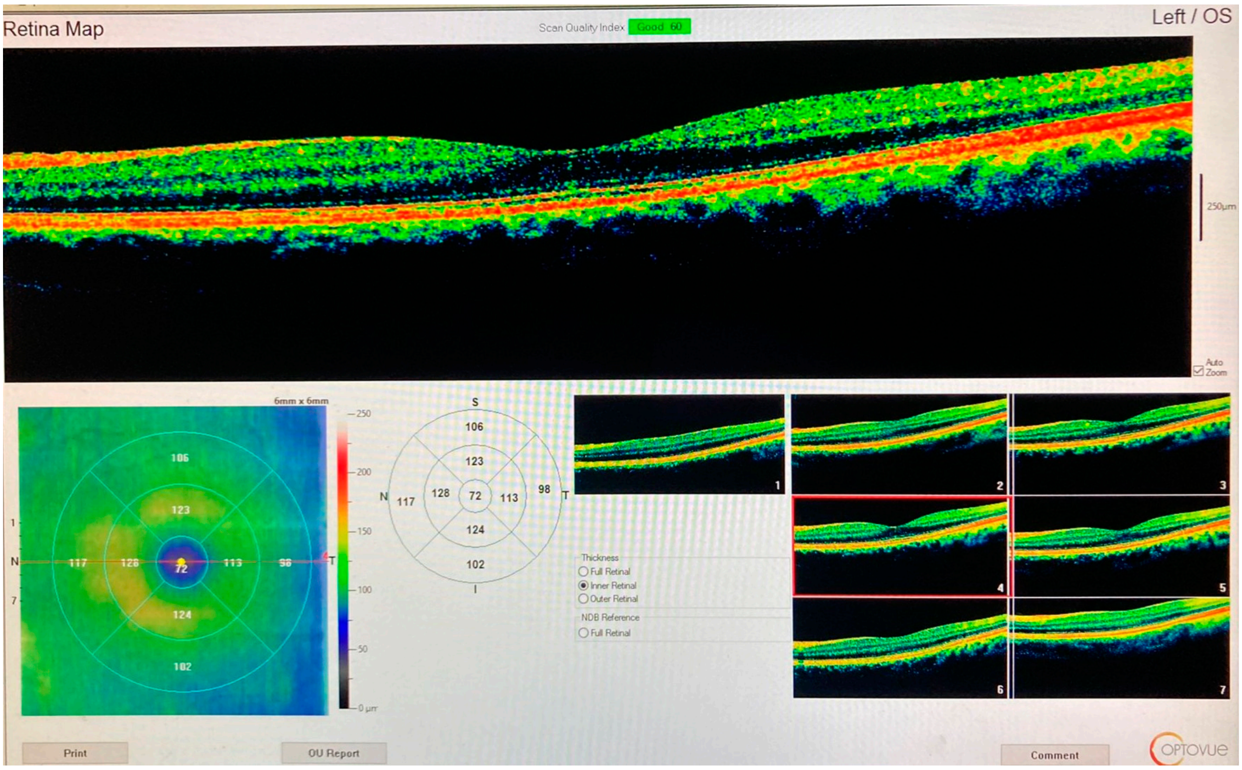

Figure S2. OCT finding of inner macular thickness (GCIPL + RNFL) across nine segments in patient with PD.

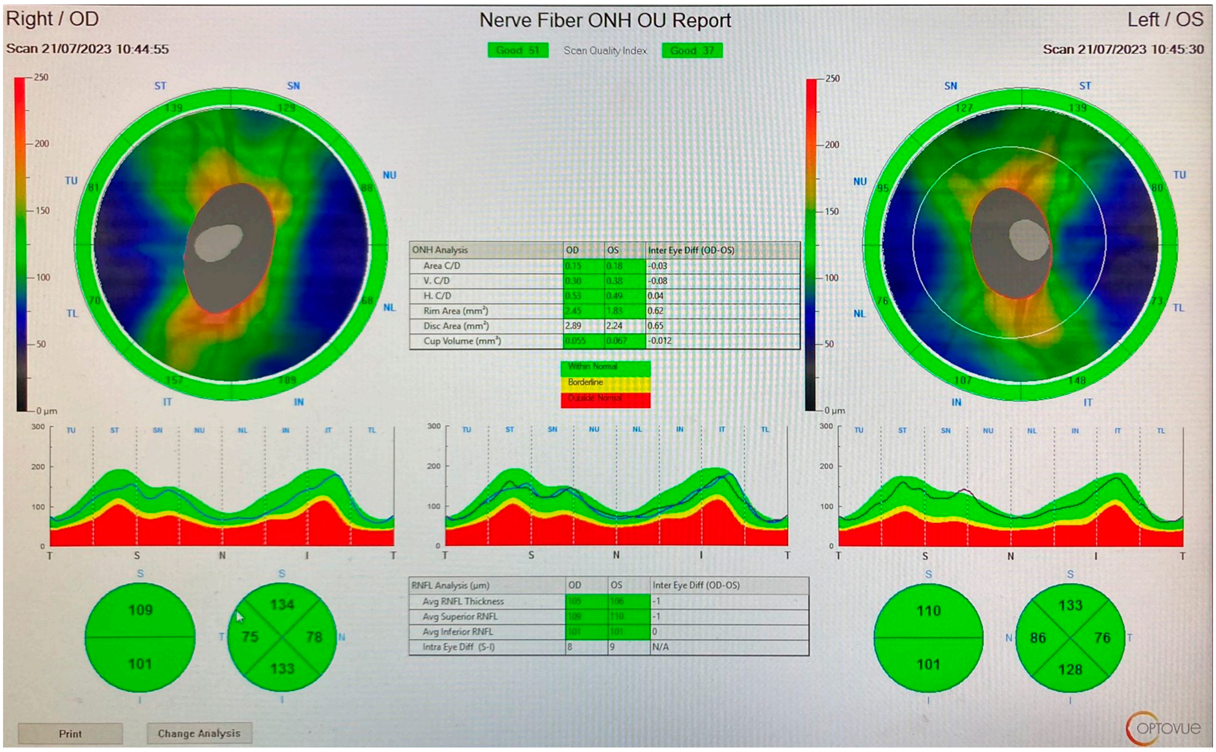

Figure S3. OCT finding of peripapillary RNFL thickness in patient with PD.
